# Mechanistic Link between Vitamin B12 and Alzheimer’s Disease

**DOI:** 10.3390/biom12010129

**Published:** 2022-01-14

**Authors:** Anna Andrea Lauer, Heike Sabine Grimm, Birgit Apel, Nataliya Golobrodska, Lara Kruse, Elina Ratanski, Noemi Schulten, Laura Schwarze, Thomas Slawik, Saskia Sperlich, Antonia Vohla, Marcus Otto Walter Grimm

**Affiliations:** 1 Experimental Neurology, Saarland University, 66424 Homburg, Germany; anna.lauer@uks.eu (A.A.L.); heike.grimm@gmx.de (H.S.G.); 2 Nutrition Therapy and Counseling, Campus Rheinland, SRH University of Applied Health Sciences, 51377 Leverkusen, Germany; Birgit.Apel@stud.srh-gesundheitshochschule.de (B.A.); Nataliya.Golobrodska@stud.srh-gesundheitshochschule.de (N.G.); Lara.Kruse@stud.srh-gesundheitshochschule.de (L.K.); Elina.Ratanski@stud.srh-gesundheitshochschule.de (E.R.); Noemi.Schulten@stud.srh-gesundheitshochschule.de (N.S.); Laura.Schwarze@stud.srh-gesundheitshochschule.de (L.S.); Thomas.Slawik@stud.srh-gesundheitshochschule.de (T.S.); Saskia.Sperlich@stud.srh-gesundheitshochschule.de (S.S.); Antonia.Vohla@stud.srh-gesundheitshochschule.de (A.V.); 3 Deutsches Institut für DemenzPrävention, Saarland University, 66424 Homburg, Germany

**Keywords:** vitamin B12, cobalamin, intrinsic factor, Alzheimer’s disease, tau pathology, Amyloid beta, homocysteine, vegetarian diet, vegan diet

## Abstract

Alzheimer’s disease (AD) is the most common form of dementia in the elderly population, affecting over 55 million people worldwide. Histopathological hallmarks of this multifactorial disease are an increased plaque burden and tangles in the brains of affected individuals. Several lines of evidence indicate that B12 hypovitaminosis is linked to AD. In this review, the biochemical pathways involved in AD that are affected by vitamin B12, focusing on APP processing, Aβ fibrillization, Aβ-induced oxidative damage as well as tau hyperphosphorylation and tau aggregation, are summarized. Besides the mechanistic link, an overview of clinical studies utilizing vitamin B supplementation are given, and a potential link between diseases and medication resulting in a reduced vitamin B12 level and AD are discussed. Besides the disease-mediated B12 hypovitaminosis, the reduction in vitamin B12 levels caused by an increasing change in dietary preferences has been gaining in relevance. In particular, vegetarian and vegan diets are associated with vitamin B12 deficiency, and therefore might have potential implications for AD. In conclusion, our review emphasizes the important role of vitamin B12 in AD, which is particularly important, as even in industrialized countries a large proportion of the population might not be sufficiently supplied with vitamin B12.

## 1. Introduction

### 1.1. Hallmarks of Alzheimer’s Disease

**Figure 1:**
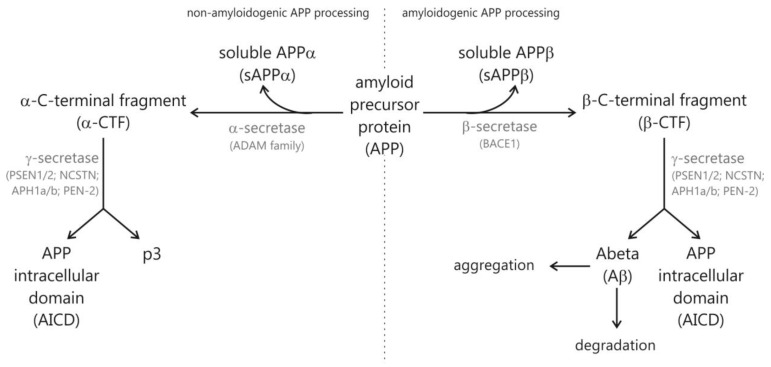
Schematic overview of amyloidogenic and non-amyloidogenic APP processing and generated cleavage products.

Alzheimer’s disease (AD) is a devastating neurodegenerative disorder and the most common form of dementia in the elderly population, clinically characterized in patients by a progressive loss of cognitive brain functions leading to memory loss and cognitive decline [1,2]. Histopathological hallmarks of AD are extracellular neuritic plaques and intracellular neurofibrillary tangles in vulnerable brain regions such as the hippocampus and cortex [3,4]. Extracellular neuritic plaques are composed of small peptides, called amyloid-β (Aβ), that are derived by sequential proteolytic cleavage of a large type-1 transmembrane protein, the amyloid precursor protein (APP) [5]. The release of Aβ peptides is strongly dependent on the amyloidogenic APP processing pathway, initiated by BACE1-mediated β-secretase cleavage of APP generating the amino-terminus (N-terminus) of Aβ peptides [6,7,8] (Figure 1). The remaining membrane-tethered carboxyl-terminal (C-terminal) APP fragment is further cleaved by γ-secretase, a heterotetrameric protein complex consisting of Presenilin 1 or 2 (PS1 or PS2), anterior pharynx defective 1 (APH-1), presenilin enhancer 2 (PEN-2) and nicastrin (NCSTN) [9,10,11]. The most abundant Aβ species generated by γ-secretase activity are Aβ40 (80–90%) and Aβ42 (10%). The relative non-specificity of γ-secretase, leading beside Aβ40 and Aβ42 to additional Aβ isoforms varying in length at the C-terminus, might be caused by the unusual intramembrane proteolytic activity of γ-secretase, cleaving APP within the hydrophobic transmembrane domain. Several lipids of cellular membranes have been found to affect the generation of Aβ peptides, Aβ aggregation and Aβ clearance [12,13,14,15,16]. The severe accumulation of Aβ peptides within brain tissue, starting years or even decades prior to the first symptoms, is considered as an important factor of AD pathogenesis, caused by an imbalance between Aβ production and Aβ clearance by Aβ-degrading enzymes such as insulin-degrading enzyme (IDE) and neprilysin (NEP) [17,18]. Beside the Aβ-releasing amyloidogenic pathway, APP can be cleaved in a non-amyloidogenic processing pathway mediated by γ-secretases. The γ-secretases have been identified as members of the ADAM (a disintegrin and metalloprotease) protein family [19,20,21] cleaving APP within the Aβ domain and thus preventing Aβ formation. Most AD cases belong to the sporadic form of AD, with a disease onset after the age of 65 (late-onset Alzheimer’s disease (LOAD)), and only approximately 5% of AD cases are caused by mutations in the genes encoding for APP or PS1/PS2 (familial Alzheimer’s disease FAD), leading to an increased production of highly amyloidogenic Aβ42 peptides. The progression of AD is classified by BRAAK stages scaled by the presence of a tau pathology through the brain. Neurofibrillary tangles beside amyloid plaques are an important pathological hallmark of AD and consist of insoluble paired helical fragments (PHF) inside neurons composed mainly of hyperphosphorylated tau proteins [4,22,23]. Tau proteins belong to the family of microtubule-associated proteins (MAPs), essential for the assembly of tubulin monomers into microtubules, to stabilize the neuronal microtubule network, important for maintaining cell shape and axonal transport [24]. The microtubule assembly promoting the activity of tau is regulated by its phosphorylation status, regulated by protein kinases [25] and protein phosphatases [26]. In AD, tau proteins are hyperphosphorylated and polymerize into paired helical fragments, forming the intraneuronal neurofibrillary tangles.

### 1.2. Risk Factors for Sporadic AD

Aging is the most important risk factor to develop LOAD. However, beside increased age, several non-genetic risk factors for LOAD are discussed, e.g., hypercholesterolemia, hyperhomocysteinemia, hypertension, atherosclerosis, diabetes mellitus and obesity [27,28]. Furthermore, dietary habits and the availability of different micronutrients have been discussed as linked to AD pathogenesis [29,30]. The possession of the apolipoprotein E (ApoE) e4 allele has been identified as the most important genetic risk factor for sporadic AD [31,32]. ApoE is one of the main lipid acceptors in the central nervous system to remove cholesterol from cells and to generate high-density lipoprotein (HDL) particles, dependent on the ApoE isoform. Beside removing cholesterol, ApoE isoforms have been found to differentially regulate Aβ clearance from the brain [33,34,35]. ApoE4 is therefore strongly associated with hypercholesterolemia, an important risk factor for AD.

Several epidemiological studies indicate that a high serum cholesterol level in midlife is associated with a higher risk for developing AD [36,37,38,39]. Cholesterol also has been reported to be elevated in the human AD post mortem brain and to be highly enriched in amyloid plaques [40,41,42]. Several cell culture studies dealing with cholesterol supplementation, cholesterol depletion or inhibition of cholesterol de novo synthesis have unambiguously illustrated that cellular cholesterol enhances Aβ production [43,44,45,46,47]. The molecular mechanisms of cholesterol-induced Aβ release out of APP can be attributed to a direct stimulation of β- and γ-secretase activity by cholesterol [45,46,47]; increased internalization of APP, leading to Aβ generation in the acidic compartments [48,49]; and a higher content of lipid rafts, which are cholesterol-rich membrane microdomains reported to be involved in amyloidogenic APP processing [43,50,51]. In addition, cholesterol has been shown to promote Aβ aggregation and Aβ toxicity [52,53,54]. Hypercholesterolemia also strongly correlates with elevated Aβ levels in several animal models [44,55,56,57,58].

Beside hypercholesterolemia, a high level of homocysteine has been discussed as a risk factor for AD [59,60,61]. Homocysteine levels have been found to be increased in cerebrospinal fluid of patients with AD compared to that of control subjects [62]. Furthermore, a meta-analysis of 13,000 AD patients compared to healthy controls revealed significantly elevated homocysteine blood levels in sporadic AD [63]. Several prospective population studies point towards elevated homocysteine levels predicting dementia up to several decades before disease onset [59,64,65]. Several pathological effects of homocysteine have been identified, including the impairment of blood–brain barrier function [66], inducing neuronal damage [67,68] and modulation of Aβ generation [69,70] and Aβ toxicity [71]. Furthermore, homocysteine generates oxidative stress, which is another risk factor for the development of AD [72,73].

Based on aging being the most important risk factor to develop AD, it has been discussed that free radicals leading to oxidative stress are involved in the pathogenesis of AD. Neurons are highly vulnerable to oxidative stress as they contain low levels of the free-radical-eliminating antioxidant glutathione [74] and high amounts of polyunsaturated fatty acids (PUFAs) that can interact with reactive oxidative species (ROS), leading to lipid peroxidation and molecular destruction [75]. In this context, it has to be mentioned that the AD protective PUFA docosahexaenoic acid (DHA) has an opposite effect on Aβ generation in its oxidized form. In the presence of oxidized DHA and lipid peroxidation products of omega-3 and omega-6 PUFAs, the soluble Aβ levels have been found to be increased [76]. Beside oxidation of lipids, increased oxidative damage to proteins as well as nucleic acids has been reported in the AD brain [77,78,79,80]. Oxidative stress thereby plays an essential role in the development of AD by promoting Aβ deposition [81,82,83,84], tau hyperphosphorylation and subsequent loss of synapses and neurons [85,86,87]. On the other hand, oxidative stress in AD can be induced by β-amyloid accumulation [85,86], hyperphosphorylated tau proteins [88,89], inflammation [90,91], metal accumulation [85,92,93] as well as mitochondrial dysfunction [85,86,94]. Interestingly, Aβ has been found to accumulate in mitochondria in AD neurons as well as in transgenic mouse models and neuronal cell cultures [95], resulting in elevated hydrogen peroxide (H_2_O_2_) production and decreased cytochrome-c oxidase activity, leading to mitochondrial dysfunction and reduced energy metabolism. Therefore, antioxidants might be potential therapeutics to prevent or treat AD. Several antioxidative substances have been reported to exert beneficial properties with respect to AD [96,97,98,99,100]. Furthermore, the fat-soluble vitamins vitamin A, D, E and K possess anti-oxidative actions and have an impact on AD [101,102,103,104]. The water-soluble vitamin B12 might be another very interesting micronutrient for AD treatment as it does not only possess anti-oxidative properties, but also interferes with different pathways reported to be involved in the pathogenesis of AD, which is discussed in the following paragraphs.

### 1.3. Vitamin B12

B vitamins, including vitamin B12 (cobalamin), are essential water-soluble micronutrients that have to be taken up in sufficient quantities from one’s diet. They are crucial for maintaining neuronal health and hematopoiesis [105]. Clinical vitamin B12 deficiency leading to myeloneuropathy or megaloblastic anemia is rare in developed countries, but subclinical vitamin B12 deficiency is common and can be found in 10 to 15% of individuals older than 60 years and in 25 to 35% of individuals aged over 80 years [105]. Subclinical vitamin B12 deficiency, defined as 119–200 pmol/L of serum vitamin B12, often remains asymptomatic over years. Based on the anti-oxidative property of vitamin B12, B12 deficiency might lead to oxidation of lipids, proteins and nucleic acids and might contribute to the development of age-related diseases, in which oxidative stress is believed to be a major factor, including AD, Parkinson disease and type 2 diabetes [106,107].

The antioxidant properties of vitamin B12 are discussed to be accomplished by different mechanisms, including direct scavenging of ROS, particularly superoxide in the cytosol and mitochondria [108,109] and indirectly stimulating ROS scavenging by preservation of glutathione [110,111]. Furthermore, vitamin B12 might protect against inflammation-induced oxidative stress by modulating cytokine and growth factor production, including interleukin-6, tumour necrosis factor alpha (TNF-α) and epidermal growth factor. Notably, the involvement of neuroinflammation is reported to play a fundamental role in the progression of AD [112,113]. A reduced vitamin B12 status is associated with an increase in interleukin-6 production and TNF-α levels [114,115], and interleukin-6 has been shown to induce hyperphosphorylation of tau [116] and TNF-α increases the Aβ burden by upregulation β-secretase production and increased γ-secretase activity [116]. Another important antioxidative mode of action of vitamin B12 is closely linked to AD: a reduction in homocysteine-induced oxidative stress. Vitamin B12 is an important cofactor of methionine-synthase, converting homocysteine into methionine. Subclinical B12 deficiency reduces the conversion of homocysteine to methionine, leading to an elevated intracellular homocysteine level [105]. Homocysteine has been discussed as mediating ROS accumulation through multiple mechanisms, including autooxidation of homocysteine, leading to H_2_O_2_, and by inhibition of cellular antioxidant enzymes, namely, glutathione peroxidase and superoxide dismutase [117]. Beside the discussed anti-oxidative function of vitamin B12, vitamin B12 exerts essential roles in the central and peripheral nervous system, maintaining the health of the nervous system [118,119], including, e.g., the cellular energetic processes, myelin, and neurotransmitter synthesis [120].

**Figure 2:**
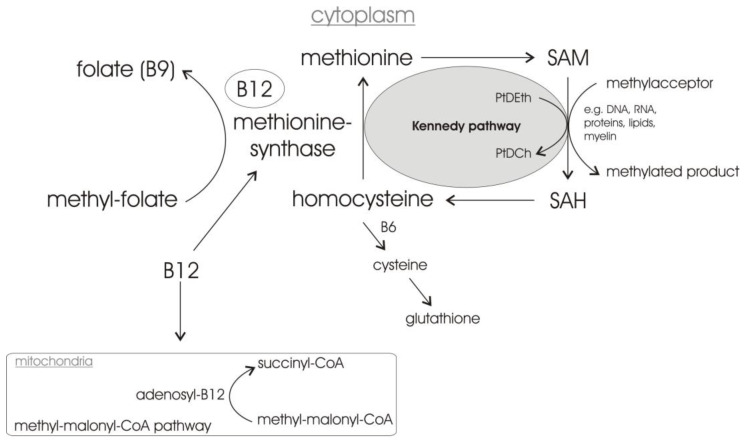
Stylized illustration of the homocysteine/methionine cycle and biochemical mechanism of action of vitamin B12 in the homocysteine/methionine and the methyl-malonyl-CoA pathway. The complex Kennedy pathway involved in phosphatidylcholine synthesis is not illustrated in detail. PtDEth: phosphatidylethanolamine; PtDCh: phosphatidylcholine; SAM: s-adenosyl-methionine; SAH: s-adenosyl-homocysteine.

As already mentioned, vitamin B12 is essential for transforming homocysteine to methionine. Homocysteine is a sulfur-containing amino acid not participating in protein synthesis. The complex biochemical pathway of homocysteine is regulated by the presence of folate (vitamin B9), vitamin B6 and vitamin B12 (Figure 2) [121,122]. Methyl-folate provides the methyl group for vitamin B12, which is in its active form—methyl-vitamin B12—an essential cofactor for the 5-methyltetrahydrofolic acid (MTHF)-dependent methionine synthase, which catalyzes the synthesis of methionine from homocysteine. Methionine is then converted to s-adenosylmethionine (SAM), a very important methyl-group donor to a variety of genomic and non-genomic substrates, e.g., DNA, RNA, proteins and lipids, being itself converted in s-adenosyl-homocysteine (SAH). A folate and/or vitamin B12 deficiency with a reduction in genomic and non-genomic methylation processes caused by folate and/or vitamin B12 deficiency, might lead to decreased DNA stability/repair and changes in gene expression/transcription, thus affecting neuronal differentiation and repair as well as promoting hippocampal atrophy and demyelination [118,123,124], impairing the propagation of action potentials. Beside the vitamin B12-dependent conversion of homocysteine to methionine, vitamin B6 enables a proportion of homocysteine to be metabolized to cysteine, a precursor of the important cellular antioxidant glutathione. In addition to the important role of vitamin B12 in the homocysteine/methionine cycle, vitamin B12 can enter the mitochondria supporting the enzyme methyl-malonyl CoA mutase (MCM), converting methyl-malonyl CoA into succinyl-CoA, an important intermediate of the Krebs/citric acid cycle, relevant for energy metabolism.

Importantly, SAM is also required for the methylation-dependent synthesis of phosphatidylcholine—the most abundant phospholipids in neuronal membranes—in the Kennedy cycle [125] (Figure 2). In this context, it has to be mentioned that supplementation with dietary precursors for lipid synthesis has been shown to increase neurite outgrowth and synaptogenesis [126,127]. Furthermore, a recent cell culture study analyzing the effect of the medical food Souvenaid, containing the specific nutrient combination Fortasyn Connect, on synaptogenesis by supplementing it with primary neuron-astrocyte co-cultures revealed positive effects. Fortasyn Connect, containing beside other supplements vitamin B12, vitamin B6 and folate, resulted in an increased number of neurons without affecting astrocyte numbers [126]. Souvenaid/Fortasyn Connect also improved the memory performances in early AD patients [128], underlining the effect of Fortasyn Connect on synaptogenesis.

Beside the above-described important roles of vitamin B12 in homocysteine/methionine metabolism, nerve metabolism (transmethylation processes), energy production and synaptogenesis, vitamin B12 is involved in fatty acid and nucleic acid synthesis. Vitamin B12 also has an impact on the formation of myelin, by affecting the DNA synthesis of myelin-producing oligodendrocytes [120,129,130,131]. Notably, recently it has been shown that myelin impairment may play an important role in AD pathology and that myelin pathology might even precede Aβ and tau pathologies of AD [132]. The regeneration of nerves after injury has also been found to be supported by vitamin B12 [119,131].

## 2. Vitamin B12 Cell Culture and Animal Studies Related to the Molecular Mechanisms of AD and AD Pathology

### 2.1. Effect of Vitamin B12 Deficiency on the Aβ Peptide Level and Aβ Deposition in AD Mice Models

In the following paragraph, animal and cell culture studies dealing with the effect of vitamin B12 with respect to AD pathology are presented. As already described in the introduction, methionine metabolism strongly depends on three important cofactors, namely, folate (vitamin B9), vitamin B6 and vitamin B12. A deficiency in these cofactors results in hyperhomocysteinemia, a risk factor for the development of AD [61]. Transgenic mice overexpressing the Swedish mutation of AD (Tg2576), leading to increased γ-secretase cleavage of APP and thus Aβ levels, fed with a diet deficient in folate, vitamin B6 and vitamin B12 for 7 months, revealed significantly elevated Aβ peptide levels in the hippocampus and cortex compared to Tg2576 fed with a control diet [133]. Immunochemical detection of Aβ deposition also showed an elevation of Aβ deposits in the hippocampus and cortex of an AD mouse model fed with a folate/vitamin B6/vitamin B12-deficient diet. Elucidating the molecular mechanism leading to the acceleration of brain amyloidosis in the diet group, Zhuo and Pratico found unchanged steady state levels of APP itself and the secretases involved in amyloidogenic APP processing, γ-secretase BACE1 and the γ-secretase components PS1 and nicastrin compared to the controls. The sAPPβ levels were also unaltered. Furthermore, reduced non-amyloidogenic processing could be excluded to be responsible for the elevated Aβ levels as unchanged levels of γ-secretase ADAM10, sAPPβ and α-CTF were found in the transgenic mice fed with a diet deficient in folate/vitamin B6 and vitamin B12. Changes in the total plasma cholesterol as the molecular mechanism for increased brain amyloidosis in the diet group is rather unlikely as total plasma cholesterol and triglycerides were not significantly different between both groups. Interestingly, α-CTF, representing the membrane-tethered fragment of γ-secretase cleavage, was significantly lower in the brains of the vitamin-deficient diet group, indicating an elevated turnover of α-CTF by γ-secretase. The authors discuss in their study that γ-secretase might be redistributed to lipid rafts, where amyloidogenic APP processing has been found to take place [134,135], in the mice fed with the vitamin-deficient diet. This would be a potential mechanism of altered γ-secretase cleavage and thus Aβ generation without affecting the total protein levels of APP or the secretases involved in its processing. Furthermore, unchanged levels of the Aβ-degrading enzymes NEP and IDE were found in this study between the diet group and the control group, indicating that the diet deficient in folate/vitamin B6 and vitamin B12 does not induce changes in Aβ catabolism [133]. Notably, the same authors found that a diet combining excessive methionine and low level of B vitamins, including folate, vitamin B6 and vitamin B12, did not alter the Aβ level and Aβ deposition in Tg2576 mice [136]. Zhuo and Pratico explain these findings by changes in the severity of diet-induced hyperhomocysteinemia. Whereas the Tg2576 mice fed with the folate/vitaminB6 and vitamin B12 diet [133] showed homocysteine levels of about 30 µM, which is in the range of homocysteine levels observed in the elderly individuals (5,5 bis 61,1 µM) [59], the TG2576 mice fed with a diet containing beside the low levels of B vitamins excessive methionine levels revealed homocysteine levels higher than 150 µM [136]. In line with the findings by Zhuo and Pratico in Tg2576 mice fed with a diet deficient in folate/vitamin B6 and vitamin B12, Fuso et al. reported elevated amyloid-β deposition in TgCRND8 mice, expressing two APP mutations—the Swedish mutation and the Indiana mutation (leading to an increase in the Aβ 42/40 ratio)—as well as wildtype (WT) littermates fed with a diet deficient in folate, vitamin B12 and vitamin B6 compared to mice fed with a control diet [137]. In both mouse strains the vitamin-deficient diet induced an elevation of PS1 and BACE expression with a more prominent effect in the TG2576 mice, which is in contrast to the findings by Zhuo and Pratico. These discrepancies in BACE1 and PS1 gene expression and protein level between the two studies, both using an AD mouse model fed with a vitamin B-deficient diet, might be caused by the use of different transgenic mouse models and the diet-induced hyperhomocysteinemia in these mice. Whereas the Tg2576 mice develop Aβ deposition at the age of 12 months, the TgCRND8 mice already develop Aβ deposition at the age of 3 months. The diet-induced hyperhomocysteinemia is also much stronger in the TgCRND8 mice, reaching a homocysteine level of about 400 µM compared to the homocysteine level of 30 µM in the Tg2576 mice. *APP* gene expression was found to be not affected by the vitamin B-deficient diet in both studies. Fuso et al. also found intraneuronal amyloid-β and a slight cognitive impairment in a water maze task at a pre-plaque stage in the TgCRND8 mice fed with the vitamin B-deficient diet [137]. Furthermore, a reduction in the ratio of SAM/SAH was found in plasma and brain of both mouse strains fed with the vitamin B-deficient diet, indicating a reduction in the methyl donor molecule SAM that donates a methyl group to different substrates, including DNA, lipids and proteins, hypothesizing that PS1 demethylation could be responsible for gene overexpression. In a follow-up study using the same mouse strains, TgCRND8 and Sv129 mice, the increase in PS1 expression could be reversed by oral supplementation of SAM at 800 mg every two days in combination with a vitamin B-deficient diet [138]. Notably, the vitamin B deprivation induced hypomethylation of specific CpG moieties in the 5′-flanking region of PSEN1 in mice and the PSEN1 promoter methylation status correlated with PS1 gene expression [138]. These findings reveal a direct relationship between B vitamin-dependent alteration of the homocysteine cycle and DNA methylation of the PSEN1 promoter, finally resulting in an elevated amyloid-β level in mice fed with a vitamin B-deficient diet.

### 2.2. Reduced Gene Expression of the Vitamin B12 Transporter Cubulin in the Intestinal Epithelium of Pre-Symptomatic Young AD Mice Models

Beside the discussed possible mechanisms of how vitamin B deficiency might lead to an elevated amyloid-β load, a recent study (2020) found that dysfunction of the intestinal epithelial barrier (IEB) occurs prior to the accumulation of brain amyloid-β and white matter injury in the central nervous system of pre-symptomatic 6-month-old Tg2576 mice [139]. Compared to 15-month-old transgenic mice that show a significant plaque burden in the subiculum and hippocampus, plaques were absent in the brain of pre-symptomatic mice at 6 months. Interestingly, significantly reduced gene expression of cubulin, a vitamin B12 transporter mediating B12 absorption in the ileum [140], in the intestinal epithelium was observed in pre-symptomatic young Tg2576 mice compared to age-matched WT littermates. This change in cubulin gene expression was not found for symptomatic 15-month-old Tg2576 mice, which showed, in accordance with a decrease in cubulin in pre-symptomatic transgenic mice, low levels of blood plasma vitamin B12. The pre-symptomatic Tg2576 mice revealing decreased expression of the vitamin B12 transporter cubulin also showed elevated levels of interleukin-9 (IL-9), vascular endothelial growth factor-α (VEGF-α) and interferon-gamma-induced protein 10 kD (IP-10) compared to age-matched littermates and symptomatic Tg2576 mice, indicating that peripheral disturbances in pro-inflammatory and angiogenic plasma cytokines occur prior to the development of cerebral pathology [139]. These results indicate that impairment of vitamin B12 intestinal absorptive function occurs before development of cerebral pathology in Tg2576 mice and potentially in human AD.

### 2.3. Vitamin B12 Supplementation Antagonizes Homocysteine Induced Changes in APP Processing and Tau Phosphorylation in Wildtype Animals

Several animal studies provide evidence that vitamin B12 supplementation exerts positive effects in respect to AD pathology not only in transgenic AD model mice but also in WT animals. Zhang et al. investigated whether vitamin B12 supplementation in hyperhomocysteinemic rats could antagonize homocysteine-induced changes in APP processing and tau phosphorylation. High plasma homocysteine levels in young WT rats (three to four months old) were induced by vena caudalis injection of homocysteine for two weeks [70]. After two weeks of homocysteine injection, elevated mRNA and protein levels of PS1 were observed, whereas the expression level of BACE1 and PS2 were found to be unchanged. Furthermore, hyperhomocysteinemic rats revealed an increase in APP phosphorylation at threonine-668, a crucial site discussed as facilitating the amyloidogenic processing of APP [141]. Along with these changes in PS1 expression and APP phosphorylation, spatial memory deficits were detected in the hyperhomocysteinemic rats [70]. The simultaneous supplementation of folate and vitamin B12 attenuated the hyperhomocysteinemic-induced changes in APP processing and improved memory in these rats. Beside changes in APP processing, these hyperhomocysteinemic rats also exhibited an AD-like tau pathology. The homocysteine-induced hyperphosphorylation of tau at multiple sites in the rat brain hippocampus [142] was attributed to inhibition of protein phosphatase 2A (PP2A) involved in the dephosphorylation of tau proteins. Again, the simultaneous supplementation of folate and vitamin B12 partially restored the plasma homocysteine level and significantly antagonized the homocysteine-induced hyperphosphorylation of tau and PP2A inactivation. The positive effect of vitamin B12 supplementation described for young three- to four-month-old hyperhomocysteinemic rats was also found for aged rats [143]. WT rats at the age of 18 month were injected with homocysteine via the vena caudalis with or without concurrent supplementation of folate/vitamin B12 for 28 weeks. Beside the homocysteine-induced inhibition of PP2A that was already found for young rats, aged homocysteinemic rats also exhibited changes in several kinases involved in tau phosphorylation: activation of glycogen synthase-3β, cyclin-dependent kinase-5, C-jun N-terminal kinase, extracellular signal-regulated kinase and activation of p38MAPK. These alterations in the activity of kinases and phosphatase PP2A also resulted in tau hyperphosphorylation and accumulation in the hippocampus and cortex in the homocysteinemic aged rats along with significant memory deficits. These biochemical and behavioral changes of chronic homocysteinemia could all be reversed by supplementation of folate/vitamin B12, indicating that folate/vitamin B12 has also positive properties in a chronic hyperhomocysteinemic rat model in reversing the AD-like tau pathology and memory deficits [143]. Although extracellular amyloid plaques and intracellular neurofibrillary tangles in the brain of individuals suffering from AD are the main pathological hallmarks of AD, impaired visual function is reported in AD patients, including retinal ganglion cell degeneration, nerve fiber layer thinning and alterations in vascular parameters. Furthermore, Aβ accumulation and tau hyperphosphorylation is present in the retina, an outgrowth of the developing brain, at early AD stages [144,145]. Supplementation of folate and vitamin B12 also revealed positive effects on Aβ level and tau hyperphosphorylation in the retina of hyperhomocysteinemic three- to four-month-old rats [146]. After homocysteine injection for two weeks the rats showed elevated Aβ42 level in the retina as well as abundant intracellular Aβ accumulation in the ganglion cell layer. This increase in Aβ pathology in the rat retina was found to be caused by a significant increase in APP, PS1 and BACE1 due to homocysteine injection. Notably, this increase in the APP, PS1 and BACE1 protein levels could be reverted by folate/vitamin B12 supplementation. Similarly, tau hyperphosphorylation present in the retina of homocysteinemic rats was rescued by folate/vitamin B12 supplementation.

### 2.4. Effect of Vitamin B12 on Amyloid Toxicity in Aβ-Expressing C. elegans as an AD Animal Model

Beside the studies revealing positive effects of vitamin B12 supplementation in transgenic mice and rats of different age, recent studies in 2021 used the roundworm *Caenorhabditis elegans* as an animal model to investigate the effects of vitamin B12 on amyloid-β toxicity [147,148]. Transgenic expression of human Aβ42 peptides in *C. elegans* body wall muscles causes AD-like pathological characteristics such as reduced ATP levels, defects in mitochondrial morphology, increased oxidative stress and a robust time-dependent paralysis [149,150,151]. Changed time to paralysis is used to identify genes or agents that influence Aβ-induced proteotoxicity. Transgenic *C. elegans* worms lacking vitamin B12 supplementation exhibited paralysis faster and more severely than worms that received vitamin B12 supplementation [147]. In-line vitamin B12 supplementation delayed Aβ-induced paralysis [148]. Along with delayed paralysis, Aβ-expressing *C. elegans* receiving a vitamin B12-containing diet showed a higher ATP level, decreased mitochondrial fragmentation and reduced oxidative species (ROS) than those without vitamin B12. Interestingly, manipulation of vitamin B12 availability during adulthood affected Aβ-induced paralysis in *C. elegans* similar to worms fed a vitamin B12-enriched diet their entire lifespan, indicating potential benefits for dietary vitamin B12 supplementation later in life. Using specific mutations in the two enzymes that need vitamin B12 as an essential cofactor, methyl-malonyl-coenzyme A mutase (*C. elegans* MMCM-1) and methionine synthase (*C. elegans* METR-1), the authors identified that vitamin B12 exerts its protective effect via the homocysteine/methionine/S-adenosylmethionine cycle, which is in line with the studies in mammals.

Several lines of evidence regarding the beneficial properties of vitamin B12 with respect to AD pathogenesis can also be found in cell culture and in vitro studies. The protective effects of vitamin B12 found in the ex vivo studies are associated to amyloid formation and fibrillization, epigenetic modifications, tau fibrillization, synaptogenesis of neuronal membranes, oxidative stress and cholesterol synthesis.

### 2.5. Vitamin B12 Inhibits Aβ Aggregation In Vitro

By the use of a Thioflavin-T fluorescent (ThT) assay to monitor Aβ aggregation, Fumo et al. could show that vitamin B12 inhibits Aβ42 aggregation in a dose-dependent manner. In the presence of 25 µM and 50 µM of vitamin B12, the ThT fluorescence intensity, reflecting Aβ aggregation, decreased to 70% and 23%, respectively compared to the control [152]. After a prolonged incubation for 70 h, vitamin B12 also significantly prevented Aβ42 from undergoing a random coil to β-sheet formation, which is closely associated with the amyloid fibril-forming tendency. Furthermore, vitamin B12 reduced the hydrophobicity of Aβ fibrils as well as the size of the aggregates. Vitamin B12 was also found to alter the fibril morphology: short and less densely populated amyloid fibrils were observed in the presence of vitamin B12. In a recent study (2021), the inhibitory effect of vitamin B12 on Aβ fibrillation could be shown by the use of artificial neuronal membranes mimicked by liposomes as Aβ generation is strongly influenced by the lipid environment of cellular membranes. To mimic neuronal cell membranes, lipid components at comparable ratios were chosen to compose the lipid vesicle: phosphatidylcholines (1,2-dimyristol-sn-glycero-3-phosphocholine), cholesterol, sphingomyelin and phosphatidylserine (L-α-phosphatidylserine) [153]. Performing a ThT fluorescent assay in the presence of Aβ1–42 and presence or absence of vitamin B12 the authors found that vitamin B12 slows down the transition from Aβ oligomers to mature fibrils and significantly reduced the content of fibrils in aqueous solution without the synthetic neuronal membranes. In the presence of synthetic neuronal membranes, the effect of vitamin B12 on Aβ fibrillization was less pronounced, but still significant. This decline in the anti-amyloidogenic properties of vitamin B12 might be due to the competitive interaction of the vitamin B12 with the lipid membrane and the Aβ peptides. However, also in the presence of synthetic neuronal membranes, vitamin B12 slowed down the Aβ fibrillization and reduced the Aβ fibril content. Beside these findings, vitamin B12 also exhibited a strong activity to disaggregate fibrils, both in an aqueous solution or in the presence of synthetic neuronal membranes, indicating that vitamin B12 is a promising target not only for AD prevention but also to cure AD.

### 2.6. Vitamin B12 Protects Cells from Cytotoxicity and Aβ-Induced Oxidative Damage

Beside the beneficial properties of vitamin B12 on Aβ fibrillization, vitamin B12 protects cells from Aβ-induced cytotoxicity and oxidative damage. In the study by Wang and Xu, PC12 cells were chronically exposed to Aβ25–35 peptides to establish an AD cell model for Aβ-induced toxicity [154]. Exposure of cells to Aβ25–35 leads to an increase in oxygen radicals, nitric oxide and disrupts calcium homeostasis, thus impairing mitochondrial function and triggering apoptosis [155,156,157]. Cotreatment of PC12 cells exposed to Aβ25–35 peptides with methyl-vitamin B12 improved cell viability by decreasing the percentage of apoptotic cells in presence of vitamin B12 (4.28% of apoptotic cells) compared to controls (7.26% of apoptotic cells) [154]. The identified mechanisms primarily underly the antioxidative function of methyl-vitamin B12 to scavenge ROS, reducing the endoplasmic reticulum-mitochondria calcium flux through IP3R (inositol-3-phosphat receptor), preventing mitochondrial dysfunction, and thus protecting cells against apoptosis and cytotoxicity. The neuroprotective antioxidative effects of vitamin B12 and the possible underlying mechanism was also addressed in H_2_O_2_-induced apoptosis in SH-SY5Y cells [158]. Treatment of SH-SY5Y cells with 200 µM H_2_O_2_ decreased cell number by 50%. Pre-treatment of SH-SY5Y cells with different concentrations of vitamin B12 (0.2, 2, 20 and 200 µM) followed by H_2_O_2_ exposure revealed that vitamin B12 promotes cell survival in a dose-dependent manner. Significant neuroprotective effects of vitamin B12 were already apparent at 2 µM vitamin B12. Protein expression profiling revealed that 22 out of 3505 proteins were significantly differentially expressed in the vitamin B12-treated cells before exposure to H_2_O_2_. The authors found that polypyrimidine tract-binding protein 1 (PTBP1) was highly associated with the protective effect of vitamin B12. Vitamin B12 exerted no protective effect on cell viability in PTBP1 knock-down SH-SY5Y cells generated by small interfering RNA. PTBP1 belongs to a subfamily of RNA-binding proteins that influence pre-mRNA processing, mRNA metabolism and transport. Therefore, the authors conclude that pre-mRNA processing is involved in the neuroprotective effects of vitamin B12, and expression of PTBP1, the main target of vitamin B12, is essential to mediate resistance against H_2_O_2_-induced oxidative damage [158]. The protective effect of vitamin B12 with respect to cell viability could be also shown in SH-SY5Y cells exposed to 70 h aged Aβ42 amyloids [152]. Cell viability of SH-SY5Y cells was decreased to 32% in presence of Aβ42 aggregates, whereas in additional presence of 25 and 50 µM vitamin B12 cell viability was increased from 32% to 74% (25 µM vitamin B12) and to 83% (50 µM vitamin B12), also indicating that vitamin B12 protects against amyloid-induced cytotoxicity.

### 2.7. Vitamin B12 Deficiency Increases the Aβ Level in Neuroblastoma Cell Lines by an Elevation in the PS1 and BACE1 Protein Level

As already found in animal studies, the DNA methylation status, regulating gene expression of genes involved in APP processing and thus Aβ generation, is affected by vitamin B12. The reduction in folate and vitamin B12 in the culture medium of two different neuroblastoma cell lines, SK-N-SH and SK-N-BE, leads to a decrease in the level of the methyl-donor SAM beside an increase in the PS1 and BACE1 protein level and an elevation in the Aβ level. Expression of APP was unaffected by folate/vitamin B12 deprivation. These results also provide evidence that DNA methylation regulates gene expression of PS1 and BACE1 and that the DNA methylation status of the promoter of these two genes is dependent on vitamin B12 [159]. The exogenous addition of SAM to the deprived medium restored the normal protein expression of PS1 and BACE1 and consequently reduced the Aβ levels [159]. Furthermore, administration of SAM in human neuroblastoma SK-N-SH cell cultures resulted in downregulated PS1 expression caused by an elevation in PS1 promoter methylation, leading to RNA downregulation and thus reduced protein synthesis, finally resulting in reduced Aβ peptide generation [160]. This is in line with the study by Fuso et al., which revealed reduced PS1 expression by addition of SAM to the neuroblastoma cell line SK-N-BE [138]. In contrast, PS1 expression was significantly elevated (3.5-fold) when SK-N-BE cells were cultured in a vitamin B-deficient medium (deficient in folate, vitamin B6 and vitamin B12). Addition of SAM to the vitamin B deficient medium restored *PS1* gene expression to that of control cells (cultured in control medium). Bisulfite modification and genomic sequencing to evaluate the methylation status of PSEN1 revealed that vitamin B deficiency induced hypomethylation of specific CpG moieties in the 5′-flanking region and that PSEN1 promoter methylation status is correlated with gene expression.

### 2.8. Vitamin B12 Inhibits Tau Polymerization by Direct Binding to Tau Proteins

Beside the findings in animal studies that vitamin B12 can inhibit tau polymerization by affecting PP2A activity [142], Rafiee et al. found that vitamin B12 inhibits tau polymerization also by direct binding to tau proteins. The authors found that vitamin B12 can bind to cysteine residues in tau and that binding to tau cysteine residues is essential for the inhibitory effect of vitamin B12 on tau fibrillization. These results indicate that binding of vitamin B12 to tau proteins, thus preventing tau aggregation, might be an alternative mechanism beside vitamin B12-induced changes in PPA2 activity regulating tau phosphorylation and tau aggregation.

### 2.9. Vitamin B12 Deficiency Increases the Cholesterol Level in Human Adipocyte Cell Cultures

Interestingly, vitamin B12 can also interfere with the biosynthesis of cholesterol, a known risk factor for AD [161,162]. Human adipocytes cultured in media containing low (0.15 nM) vitamin B12 or no (0 nM) vitamin B12 were compared to control cells incubated with 500 nM B12 (representing adequate vitamin B12). Total cholesterol was significantly increased in human adipocytes exposed to low or no vitamin B12 conditions compared to the controls [163]. qPCR analysis revealed that several genes involved in cholesterol de novo synthesis, including the rate-limiting enzyme 3-hydroxy-3-methylglutaryl-CoA reductase (HMGCR), were significantly increased in vitamin B12-reduced or -deficient cells. Furthermore, low vitamin B12 significantly elevated the gene expression of the sterol regulatory element-binding proteins (SREBP1 and 2) as well as the sterol regulatory element-binding transcription factors (SREBF1 and 2), involved in the regulation of cholesterol synthesis and gene expression of the low-density lipoprotein receptor (LDLR). The authors found that the induction of cholesterol biosynthesis in cells with insufficient vitamin B12 was associated with a significant decrease in SAM, involved in DNA methylation. Genome-wide and targeted DNA methylation analysis revealed that the promoter regions of SREBF1 and LDLR were hypomethylated under vitamin B12-deficient conditions, leading to increased expression and thus cholesterol synthesis. Beside the increased expression of BACE1 and PS1 [70,137,138,159,160] in vitamin B12-deficient cells or animals, thus leading to elevated Aβ levels, this study indicates that vitamin B12 deficiency elevates Aβ generation by increasing the amount of cholesterol, known to elevate Aβ generation [43,45,47,55].

**Figure 3:**
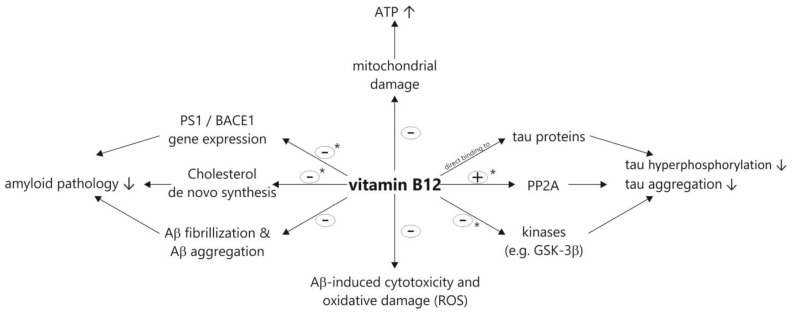
Summary of the proposed beneficial properties of vitamin B12 with respect to amyloid and tau pathology in AD based on the discussed cell culture, in vitro and animal studies. In this context it has to be mentioned that some of the illustrated potential mechanisms (marked with asterisks) are based on studies under vitamin B12 (and folate) deficiency/hypovitaminosis.

Figure 3 illustrates the potential beneficial properties of vitamin B12 on the pathological processes of AD based on the discussed animal, cell culture and in vitro studies.

## 3. Clinical Studies

Several clinical randomized controlled trials showed beneficial effects of vitamin B12 alone or in combination with for example other B vitamins or folic acid. In the following paragraph we will present recent clinical studies examining a potential link between vitamin B12 and AD. This was due to the detection of the vitamin B12 status in mild-cognitive impairment (MCI) and AD patients or observing the effect of vitamin B12 supplementation. Firstly, studies including elderly adults without cognitive decline or MCI patients will be presented, separating those using combinations of vitamin B12 and other supplements from those using vitamin B12 alone (Table 1). Secondly, clinical trials and meta-analysis of vitamin B12 and AD-diagnosed patients will be discussed (Table 2).

In a recent study dealing with a potential role of paraoxonase 1 (PON1), a high-density lipoprotein-associated enzyme, in the development of neurological diseases, the authors could show that B vitamins abrogated associations of PON1 with cognition. A total of 95 individuals with MCI received a daily dose of folic acid (0.8 mg), vitamin B12 (0.5 mg) and B6 (20 mg) in this randomized, double-blind placebo-controlled trial and 101 MCI patients received the placebo for a period of two years. A significant association of the phenylacetate hydrolase activity of PON1 with global cognition, verbal episodic memory and attention/processing speed at the end of the study was found in the placebo group. In the intervention group, B vitamins ameliorate the detrimental effects of PON1 on cognition. This study highlighted a novel positive aspect of B vitamin treatment on the central nervous system [164]. In contrast, a randomized controlled trial from 2010, investigating the effect of supplementation with daily doses of 2 mg folic acid, 25 mg vitamin B6 and 500 µg vitamin B12 over two years, did not detect any beneficial effects of B vitamins on cognitive function or the risk of cognitive impairment or dementia. However, as the authors stated, a limitation of this study could be the selection of the participants, since the men aged ≥ 75 years with preexisting hypertension were not selected based on high homocysteine levels or low vitamin serum concentrations and this could have compromised the effect size of the intervention [165]. Contrary to these findings, numerous further studies provide evidence for an association between B vitamins and cognitive functions. For example, a meta-analysis including 21 observational studies aimed to examine the association between the intake and plasma levels of vitamins B12, B6 and folate and the prevention of cognitive decline in community-dwelling older adults aged ≥ 45 years. This study reported higher levels of vitamin B12 to be associated with better cognition in cross-sectional studies (odds ratio = 0.68, 95% confidence interval = 0.51–0.90), but not in sensitivity analyses or prospective studies [166]. Furthermore, a recent meta-analysis (2021) also reported a preventive efficacy of vitamin B supplements on the cognitive decline of elderly adults. The analyzed 21 randomized controlled trials involving 7571 participants revealed a significant effect in global cognitive function and homocysteine. This effect is lacking in parameters of information processing speed, episodic memory, and executive function. Based on this, the authors recommend vitamin B supplements to be considered as a preventive medication to MCI patients since vitamin B might delay or maintain the cognitive decline of elderly adults [167]. In line with this evidence of the beneficial effects of vitamin B12 in individuals without or with mild cognitive impairments, a recent randomized controlled trail reported that the combination of oral vitamin B12 (25 µg) and folic acid (800 µg) for six months reduced the levels of peripheral inflammatory cytokines and improved cognitive performance significantly in MCI patients, assessed by the measurement of the full-scale intelligence quotient (IQ), verbal IQ as well as information and digit span scores. Interestingly, the combined intervention with vitamin B12 and folic acid was significantly advanced compared to either vitamin B12 or folic acid alone for all endpoints [168].

Besides inflammation and cognition, further studies show that treatment with vitamin B is also able to prevent brain atrophy of the key regions related to cognitive decline in MCI patients. Scientists from the University of Oxford obtained numerous findings in this context from their single-center, randomized, double-blind controlled trial (VITACOG trial) of daily high-dose B vitamins treatment (0.8 mg folic acid, 20 mg vitamin B6, and 0.5 mg vitamin B12) of individuals with MCI for two years. They used serial volumetric magnetic resonance imaging scans to evaluate the change in the rate of atrophy of the whole brain. Significantly reduced rates of brain atrophy per year in the treated group compared to the placebo group (0.76% vs. 1.08%, *p* = 0.001) were demonstrated. Moreover, this treatment response was found to be related to the baseline homocysteine levels [169]. As a secondary outcome of this study, a significant benefit of vitamin B intervention in MCI-suffering individuals, with higher baseline homocysteine levels in global cognition, episodic memory and semantic memory, has been reported [170]. Additionally, the authors reported a seven-fold reduced gray matter atrophy by this combined B vitamins treatment over two years. This beneficial vitamin B effect was restricted to participants with high homocysteine and based on this the authors conclude that B vitamins reduce homocysteine, which directly lower gray matter atrophy and thereby slowing cognitive decline [171]. The same authors could show in a following study that plasma omega-3 fatty acid concentrations modify the vitamin B effect on brain atrophy rates in elderly people with MCI. A total of 85 MCI patients were treated daily with high-dose vitamin B supplementation (0.8 mg folic acid, 20 mg vitamin B6, 0.5 mg vitamin B12) for two years. In subjects with high baseline omega-3 fatty acids (>590 µmol/L), this slowed the mean atrophy rate significantly by 40% compared with placebo-treated participants. Through this study, the importance to identify the subgroups likely to benefit in clinical studies was highlighted [172]. In their recent randomized controlled trial, these authors could show that the baseline omega-3 fatty acid status interacts with the effects of vitamin B treatment in individuals with MCI. They found the final scores for the verbal delayed recall, global cognition and clinical dementia rating (CDR) sum-of-boxes improved in the MCI participants randomized to B vitamins (folic acid, vitamin B6 and B12) for two years according to increasing baseline concentrations of omega-3 fatty acids. In more detail, higher docosahexaenoic acid concentrations alone significantly enhanced the beneficial cognitive effects of B vitamins. Based on this, a combined supplementation of B vitamins and omega-3 fatty acids is suggested as potential therapy to slow the conversion from MCI to AD, which should be analyzed in further studies [173].

An earlier randomized control trial examined the effect of a nutraceutical formulation (NF) containing vitamin B12, folate, alpha-tocopherol, S-adenosyl methionine, N-acetyl cysteine and acetyl-L-carnitine on cognitive performance in MCI patients. In the first six months of the study, the 34 individuals were randomized to NF or the placebo and in a six-month open-label extension all individuals received NF. The intervention resulted in improvements in the Dementia Rating Scale and maintenance of the baseline performance in CLOX-1. These beneficial effects could not be observed in the placebo group, only during the open-label extension [174].

Moreover, a randomized control trial performed among 299 men (≥75 years) with daily treatment of 2 mg folate, 25 mg vitamin B6 and 400 µg vitamin B12 over two years reported an influenced plasma level of Aβ40. The mean increase of Aβ40 was 7.0 pg/mL in the intervention group compared to 26.8 pg/mL in the placebo group. Based on these data, the authors suggested a potential role of B vitamins in the prevention of AD [175]. In contrast to the Aβ40 levels, the degree of immune activation and inflammation seems to remain unchanged due to vitamin B supplementation, as a clinical trial from 2006 reported. The authors examined the effects of daily vitamin B supplementation (50 mg vitamin B1, 50 mg vitamin B6, 5 mg folic acid and 0.05 mg vitamin B12) on the homocysteine and neopterin concentrations in 58 patients with AD (n = 30), vascular dementia (n = 12) and MCI (n = 16) after one month. While the homocysteine concentrations declined significantly after one month of vitamin B supplementation, the concentrations of neopterin were not influenced. Since analysis of the neopterin concentrations is used to monitor mediated immune activation and inflammation status, these data suggested that B vitamin supplementation did not influence the immune system activation status [176].

In the prospective analysis of a recent clinical trial with participants between 50 and 70 years of age, an inadequate dietary vitamin B12 uptake was significantly associated with an accelerated cognitive decline. Moreover, the authors were able to show in MCI patients that reduced serum vitamin B levels may contribute to worse cognitive performance by affecting the DNA methylation levels of redox-related genes such as *NUDT15* or *TXNRD1* [177]. In line with this, a controlled clinical trial, including 28 nursing home residents with dementia and low serum vitamin B12 levels (<250 pg/mL) and 28 participants with normal serum vitamin B12 levels (>300 pg/mL), reported significant improvement in metabolic and hematologic parameters after 16 weeks of intramuscular vitamin B12 treatment (1000 µg daily for one week, then 1000 µg weekly for 15 weeks). However, the authors could not detect beneficial effects on cognitive or psychiatric symptoms mediated by the vitamin B12 supplementation, which could be explained due to the short follow-up period of 16 weeks [178].

**Table 1:** Clinical studies examining a potential link between vitamin B12 and cognitive performance in elderly adults without cognitive impairments or MCI patients. MCI: mild cognitive impairment. RCT: randomized controlled trial. SAM: S-adenosyl methionine. NAC: N-acetyl cysteine. ALCAR: acetyl-L-carnitine.

Author	Year	Type of Study/Duration/n	Main Finding
Perla-Kaján et al. [164]	2021	RCT/2 years/intervention group (n = 95) and placebo group (n = 101)	A daily dose of folic acid, vitamin B12 and B6 ameliorates detrimental effects of paraoxonase 1 (PON1) on cognition in individuals with mild cognitive impairment
Li et al. [167]	2021	Meta-Analysis/until 1 December 2019/21 RCTs (7571 participants)	Vitamin B supplements (vitamin B12, B6, folic acid alone or in combination) show preventive efficacy on cognitive decline of elderly adults
Zhang et al. [166]	2020	Meta-Analysis/until 8 August 2019/21 observational studies (sample sizes: 155–7030)	Higher levels of vitamin B12 concentration were associated with better cognition in cross-sectional studies
Ma et al. [168]	2019	RCT/6 months/240 participants with MCI (four treatment groups)	Daily oral uptake of vitamin B12 (25 µg) in combination with folic acid (800 µg) significantly improved cognitive performance and reduced inflammatory cytokine levels in peripheral blood in MCI elderly
Oulhaj et al. [173]	2016	RCT/2 years/266 participants with MCI aged ≥70 years	The effect of vitamin B treatment on cognitive decline in MCI depends on the omega-3 fatty acid concentrations
Remington et al. [174]	2015	RCT/6 months nutraceutical formulation (NF) and placebo + 6 months extension with NF for all participants/34 individuals with MCI	Intervention with nutraceutical formulation (400 µg folic acid, 6 µg B12, 30 I.U. alpha-tocopherol, 400 mg SAM, 600 mg NAC, and 500 mg ALCAR) improved cognitive performance
Jernerén et al. [172]	2015	RCT/2 years/intervention group (n = 85) and placebo groups (n = 83)	High plasma long-chain omega-3 fatty acids are important for the beneficial effect of vitamin B treatment (folic acid, vitamin B6 and B12) on brain atrophy in MCI patients
Douaud et al. [171]	2013	RCT/2 years/intervention group (n = 80) and placebo group (n = 76)	High-dose vitamin B treatment (folic acid, vitamin B6 and B12) slow the atrophy of specific brain regions related to AD and cognitive decline in MCI patients
A de Jager et al. [170]	2012	RCT/2 years/intervention group (n = 133) and placebo group (n = 133)	Vitamins B (folic acid, vitamin B6 and B12) appear to slow cognitive and clinical decline in MCI patients, especially among participants with elevated baseline homocysteine levels
Ford et al. [165]	2010	RCT/2–8 years/299 hypertensive men ≥ 75 years	No beneficial effect of supplementation with B vitamins (B12, B6, folic acid) on cognitive function (2 years outcome) or the risk of cognitive impairment or dementia (8 years outcome)
Smit et al. [169]	2010	RCT/2 years/intervention group (n = 85) and placebo group (n = 83)	Accelerated brain atrophy in MCI patients can be slowed by treatment with B vitamins (folic acid, vitamin B6 and B12)
Flicker et al. [175]	2008	RCT/2 years/intervention group (n = 150) and placebo group (n = 149)	Reduced increase of plasma Aβ40 levels in older men treated with a combination of folate, vitamin B6 and B12 compared to placebo group
Frick et al. [176]	2006	Clinical Trial/1 month/58 patients (AD, n = 30; vascular dementia, n = 12; MCI, n = 16)	Daily supplementation of B vitamins (vitamins B1, B6, B12, folic acid) declines concentrations of homocysteine but not of neopterin in demented patients
An et al. [177]	2019	Clinical trial/2.3 years/2533 participants for longitudinal study + a subgroup of 109 MCI patients and 73 controls for DNA methylation and biochemical analyses	Significant association between inadequate dietary intake of vitamin B12 and accelerated cognitive decline, which may be mediated by affected methylation levels of specific redox-related genes
Van Dyck et al. [178]	2009	Controlled clinical trial/16 weeks/replacement group with low serum B12 levels (n = 28) and control group with normal serum B12 levels (n = 28)	Vitamin B12 replacement in dementia with low serum B12 levels resulted in significant improvements in hematologic and metabolic parameters but is unlikely to benefit cognitive or psychiatric symptoms

Besides these clinical studies investigating the role of vitamin B12 in elderly without cognitive decline or in MCI patients, numerous recent trials aimed to analyze this link in patients suffered from AD. In a meta-analysis from 2015, it could be demonstrated that AD patients have lower levels of vitamin B12 in plasma than healthy individuals. Interestingly, these differences in vitamin B12 levels were further enlarged with increased age [179]. These significantly lowered plasma levels of vitamin B12 in AD patients are in line with the findings of a meta-analysis published one year before [180] or an earlier study reporting that the median vitamin B12 concentration was reduced in neurological patients (AD: n = 34; Parkinson’s disease: n = 46; other cognitive disorders: n = 47) compared to healthy control individuals [181].

In a pilot study of 69 AD patients supplemented with a vitamin B12 and B6 combination for eight weeks, the authors reported a significant reduction in fasting and post-methionine-loading homocysteine. Interestingly, these reductions were also found in AD patients taking standard multivitamin supplements [182]. In line with these findings, a randomized controlled trial, including male and female patients with mild to moderate AD, found decreased homocysteine concentrations after 26 weeks of supplementation with a multivitamin supplement containing vitamins B6, B12 and folic acid. Besides 500 mg mecobalamin (B12), 5 mg pyridoxine (B6), 1 mg folic acid, other vitamins and iron, all participants received an acetylcholinesterase inhibitor in this study, which aimed to investigate if oral multivitamin supplementation would improve cognitive function and reduce serum homocysteine levels in AD patients. Under the conditions used in this trial, no statistically significant beneficial effects of this intervention on cognition or performance of activities of daily living could be observed [183]. An additional randomized controlled trial, which was published one year later (2008), reported similar findings of high-dose vitamin B supplementation and cognitive decline in AD. A total of 202 individuals with mild to moderate AD received 5 mg folate, 25 mg vitamin B6 and 1 mg vitamin B12 daily, while 138 individuals were treated with an identical placebo for 18 months. In line with the previously described study, the vitamin B intervention was effective in reducing the homocysteine levels, but no beneficial effects on cognitive measurements were observed [184]. In line with this, a meta-analysis including four randomized controlled trials reported supplementation of folic acid along with vitamin B12 and/or vitamin B6, resulting in decreased serum homocysteine levels, but did not influence cognitive improvement—as evaluated by a mini-mental state examination (MMSE)—in patients with cognitive decline secondary to AD or dementia [185].

A pilot study examining the efficacy of a vitamin/nutraceutical formulation (NF) (400 µg folic acid, 6 µg vitamin B12, 30 IU vitamin E, 400 mg S-adenosyl methionine, 600 mg N-acetyl cysteine and 500 mg acetyl-L-carnitine) for 12 months in 14 participants with clinically diagnosed early-stage AD reported improved cognitive functions, for example, in the Dementia Rating Scale and clock-drawing tests (Clox 1 and 2) [186]. In the following randomized controlled trial, the efficacy of this formulation (NF) was analyzed in twelve institutionalized patients with a moderate-stage to later-stage AD diagnosis. After three months of daily intake, a clinically significant delay in decline in the Dementia Rating Scale and clock-drawing test could be observed compared to the placebo group [187]. Later, a double-blind, multi-site, phase II study of this nutritional formulation for cognition and mood in AD was performed, including 106 AD patients for a duration of up to six months with an open-label extension with NF supplementation for six additional months. This study extended the phase I studies, showing a maintained or improved cognitive performance as well as mood and behavior [188]. One year later, the same authors could show that this nutraceutical formulation causes that the 24 individuals diagnosed with AD, which received this supplementation for 12 months under open-label conditions, maintained their baseline cognitive performance and behavioral and psychological symptoms of dementia [189].

An additional clinical trial investigating the effect of vitamin B12 in combination with other components aimed to investigate the treatment of AD with the cholinesterase inhibitor donepezil combined with the most common antioxidants in a so called formula F (100 mg Carnosine, 1.4 mg vitamin B1, 1.6 mg vitamin B2, 28 mg vitamin B3, 2 mg vitamin B6, 200 µg vitamin B9, 1 µg Cyanocobalamin (B12), 30 mg vitamin C, 20 mg vitamin E, 10 mg Coenzyme Q10, 800 RE β-carotene, 27.5 µg selenium, 10 mg L-cysteine and 25 mg Ginkgo biloba). A total of 52 patients suffering from moderate AD, who already received 5 mg donepezil per day for at least two months, were divided into two groups and followed for a period of six months. The MMSE II score, which was measured as secondary parameter to evaluate the overall clinical condition, was significantly improved in patients treated with donepezil plus formula F [190]. Moreover, the beneficial effects of the combined treatment with antipsychotic drugs and vitamin B12 with respect to pro- and anti-inflammatory cytokines were reported in AD patients. Besides reduced expressions of IL-8 and TNF-α, and an elevated expression of TGF-β, the combination of vitamin B12 and quetiapine decreased the pain in psychotic AD patients [191]. A further clinical trial, aiming to assess the influence of vitamin B supplementation on parameters of oxidative stress, inflammation and cognition in AD and MCI patients, reported significantly decreased levels of carbonyl proteins in patients supplemented with vitamin B1 (50 mg), B6 (50 mg), B12 (0.05 mg) and folic acid (5 mg) for three months. Additionally, a negative correlation between carbonyl proteins and MMSE was found, suggesting carbonyl proteins as potential markers for the monitoring of patients with dementia [192].

The LipiDiDiet trial examined the use of Souvenaid, containing Fortasyn Connect, comprising docosahexaenoic acid, eicosapentaenoic acid, uridine monophosphate, choline, phospholipids, selenium, folic acid, vitamin B12, B6, C, and E in prodromal and early stages of AD. No significant effect of this non-pharmacological intervention on the primary efficacy endpoint, change over 24 months in a composite score of cognitive performance evaluated by a neuropsychological test battery, was observed. But the authors reported significant benefits in parameters of disease progression, like in attention, memory, executive function (domains of cognition affected in AD) and hippocampal atrophy [193]. Moreover, in preceding studies the influence of Fortasyn Connect on nutritional markers and levels of plasma homocysteine could be shown [194,195]. These findings suggested this intervention as beneficial for earlier stages of AD since risk factors for its progression were affected.

A recent randomized controlled phase II clinical study investigated the efficacy of BrainUp-10^®^ in modifying behavioral and cognitive symptoms as well as in providing life quality in patients with mild to moderate AD. Besides significantly reduced homocysteine levels, the authors reported significant improvements in the MMSE score after 24 weeks of daily BrainUp-10^®^ treatment in 82 AD patients. Moreover, scores of the Neuropsychiatry Index, caregiver distress and alimentary response improved significantly after twelve weeks. Additionally, apathy was significantly reduced both after four and twelve weeks. Since no adverse events were observed, this nutraceutical may enable early-stage AD patients to receive the benefits in cognition and behavior [196].

A further randomized controlled study from 2021 examined the effects of a combined supplementation of folic acid and vitamin B12 on cognitive impairment and inflammation in AD patients. A total of 51 participants received 1.2 mg folic acid and 50 µg vitamin B12 daily for six months and 50 participants were in the placebo group. Compared to the untreated subjects, beneficial effects in the Montreal Cognitive Assessment (MoCA) total, naming, orientation and Alzheimer’s Diseases Assessment Scale—Cognitive subscale (ADAS-Cog) score of attention were observed in the intervention group. Moreover, positive effects in plasma SAM, SAM/SAH, SAH and serum homocysteine and TNF-α resulted from this intervention [197].

**Table 2:** Clinical studies dealing with vitamin B12 and Alzheimer’s disease. RCT: randomized controlled trial. MCI: mild cognitive impairment. SAM: S-adenosyl methionine. NAC: N-acetyl cysteine. ALCAR: acetyl-L-carnitine.

Author	Year	Type of Study/Duration/n	Main Finding
Chen et al. [197]	2021	RCT/6 months/intervention group (n = 51) and placebo group (n = 50)	Supplementation of folic acid and vitamin B12 had a beneficial therapeutic effect in AD patients who were not on a folic acid-fortified diet
Guzman-Martinez et al. [196]	2021	RCT/24 weeks/82 mild to moderate AD patients	The nutraceutical BrainUp-10^®^, containing vitamin B12, produces a significant improvement in apathy, ameliorating neuropsychiatric distress of patients
Rasmussen [193]	2019	RCT/24 + 12 months/311 patients with prodromal AD	Fortasyn Connect, a multi-nutrient combination containing vitamin B12, may show benefit on domains of cognition affected by AD
Vakilian et al. [191]	2017	Clinical trial	Vitamin B12 in combination with antipsychotic drugs is able to reduce and induce the expression of pro- and anti-inflammatory cytokines in AD patients
Zhang et al. [185]	2017	Meta-Analysis/until 7 May 2015/4 studies included	Data on vitamin B-induced improvement in cognition by reducing homocysteine levels are conflicting and should be addressed in further studies
Remington et al. [189]	2016	RCT/12 months/24 individuals diagnosed with AD	Over the duration of nutraceutical formulation (folate, alpha-tocopherol, vitamin B12, SAM, NAC, ALCAR) supplementation behavioral and psychological symptoms of dementia as well as baseline cognitive performance were maintained
Remington et al. [188]	2015	Clinical trial/3- or 6-months intervention + 6 months open-label extension/106 individuals with AD	The results of this trial extended phase I studies showing maintained or improved cognitive performance and mood/behavior after supplementation of nutraceutical formulation (folate, alpha-tocopherol, vitamin B12, SAM, NAC, ALCAR) in AD patients
Rommer et al. [192]	2016	Clinical trial/3 months/healthy control (n = 15), AD or MCI (n = 16), supplemented AD or MCI (n = 17)	Supplementation of vitamins B1, B6, B12 and folic acid for three months resulted in decreased levels of carbonyl proteins, which negatively correlated with MMSE in AD/MCI patients
Shen et al. [179]	2015	Meta-Analysis/up to January 2014/68 studies included	Higher homocysteine and lower folic acid and vitamin B12 levels in AD patients than healthy individuals
Lopes da Silva et al. [180]	2014	Meta-Analysis/literature published after 1990/more than five publications for a specific nutrient	Significantly lower plasma levels of vitamin B12 were found in AD patients.
Cornelli [190]	2010	Clinical trial/6 months/52 moderate AD patients already being treated with 5 mg donepezil per day for at least two months	Treatment with formula F (Carnosine, vitamins B1, B2, B3, B6, B9, B12, C, E, Coenzyme Q10, β-carotene, selenium, L-cysteine, Ginkgo biloba) decreased oxidative stress and homocysteine levels and improved MMSE II scores significantly
Remington et al. [187]	2009	RCT/9 months/12 institutionalized patients diagnosed with moderate-stage to later-stage AD	Supplementation of a vitamin/nutraceutical formulation containing folate, vitamin B12, alpha-tocopherol, S-adenosyl methionine (SAM), N-acetyl cysteine (NAC), acetyl-L-carnitine (ALCAR) seems to delay the decline in cognition, mood, and daily function
Chan et al. [186]	2008	Clinical trial/12 months/14 community-dwelling individuals with early-stage AD	Treatment with a vitamin/nutraceutical formulation (folate, vitamin B12, alpha-tocopherol, SAM, NAC, ALCAR) resulted in improved cognitive performance
Aisen et al. [184]	2008	RCT/18 months/intervention group (n = 202) and placebo group (n = 138) of AD patients	Daily supplementation of folate, vitamin B6 and B12 for 18 months was effective in reducing homocysteine levels, but not in slowing cognitive decline in individuals with mild to moderate AD
Sun et al. [183]	2007	RCT/26 weeks/89 patients with mild to moderate AD and normal folic acid and vitamin B12 concentrations	Multivitamin supplement including vitamins B12, B6 and folic acid reduced concentrations of homocysteine but had no statistically significant beneficial effects on cognition compared to placebo treatment
Aisen et al. [182]	2003	Clinical trial/8 weeks/69 subjects with AD, including 33 with standard multivitamin supplements	This open-label trial shows high-dose, combined vitamin B12 and B6 supplementation to reduce homocysteine levels in AD patients
Teunissen et al. [181]	2003	Clinical trial/one-point/neurological patients (AD: n = 34; Parkinson’s disease: n = 46; other cognitive disorders: n = 47) and healthy controls (n = 61)	Compared to healthy individuals the median vitamin B12 concentration was decreased in all neurological patients

## 4. Is There an Association of Diseases and Medications, Known to Be Linked to Vitamin B12 Deficiency, with AD?

In a further step of our review, we aimed to analyze if diseases and medications, which are known to be linked to vitamin B12 deficiency, can also be associated with dementia, especially AD (Table 3).

An example of a disease associated with a vitamin B12 deficiency is inflammatory bowel disease (IBD). Among patients with IBD, deficiencies of micronutrients, such as vitamin B12, are common. Crohn’s disease patients are more affected than ulcerative colitis patients [198]. Possible causes for this IBD-related vitamin B12 deficiency could be ileal disease or resection, fistulas and small bowel bacterial overgrowth, amongst others. Up to 22% of Crohn’s disease patients were reported to be affected by reduced vitamin B12 serum levels [199,200]. Moreover, a recent study also reported significantly higher rates of vitamin B12 deficiency in Crohn’s disease compared to ulcerative colitis patients and reduced deficiencies after a six-months treatment with vitamin B12 [201]. In this context, it must be mentioned that evaluating the vitamin B12 status based on serum vitamin B12 levels is relatively insensitive. Holotranscobalamin combined with methylmalonic acid is suggested to be a more accurate way to identify an impaired vitamin B12 status [202]. A recent clinical study analyzed if there is an association between IBD and the risk of dementia in patients aged over 60 years with an initial diagnosis of Crohn’s disease or ulcerative colitis (n = 3850) and patients without IBD (n = 3850) over a period of 15 years. The authors reported that IBD is associated with a 1.22-fold elevated risk of developing dementia [203]. In line with these findings, a longitudinal study, also published in 2021, reported IBD to be associated with a higher risk of dementia. Moreover, the greatest increase was observed in the risk of developing AD after 16 years [204]. Based on these recent results, the relationship between IBD and dementia should be the aim of future research. For example, a long-term supplementation of IBD patients with vitamin B12 followed by an evaluation of a dementia/AD diagnosis should be performed.

A further disease, which is considered as a possible cause for the deficiency in water-soluble vitamin B12, is gastritis [205]. It can be differentiated between environmental atrophic gastritis, which can be caused by *Helicobacter pylori*, environmental factors or specific diets, or autoimmune atrophic gastritis [206]. A recent study (2021) demonstrated an association of atrophic gastritis (AG) with significantly lower serum total vitamin B12 levels compared to individuals without AG. A possible explanation for this finding could be that this disease mediates the suppression of gastric acid and may thereby impair the absorption of vitamin B12 from foods [207]. In line with this, an earlier study also reported a prevalence of 2.5% of low serum vitamin B12 levels related to atrophic corpus gastritis [208]. Further authors aimed to investigate the association between gastritis and dementia in older adults and found an increased prevalence of dementia in individuals suffering from gastritis compared to healthy controls (29.5% vs. 13.2%) [209]. Moreover, an adjusted, significant odds ratio of 2.42 was found for gastritis associated with dementia. A recent Swedish study reported an elevated risk of an AD diagnosis in patients previously diagnosed with an autoimmune disorder. An increase in the standardized incidence ratio of 1.64 was reported for pernicious anemia [210]. Pernicious anemia (PA) is defined as a macrocytic anemia, which is one of the distinctive manifestations of autoimmune metaplastic atrophic gastritis and caused by vitamin B12 deficiency [211]. In line with the findings of this recent study, an early study could also associate neuropsychiatric conditions such as dementia with PA [212]. Besides the suggested vitamin B12 deficiency-mediated associations, a link between another cause of gastritis and AD also was shown, that of *Helicobacter pylori*. In a cohort of 50 AD patients, 88% (44 out of 50 participants) showed a histologically proven infection with *H. pylori* compared to 46.7% (14 out of 30 participants) in the control group. Proving the causality of this association by eradicating *H. pylori* and observing the course of AD should be the aim of further research [213].

Besides diseases, also surgical interventions, such as a total or partial gastrectomy, could cause a severe vitamin B12 deficiency [205]. A study comparing the risk of AD in gastric cancer patients who underwent gastrectomy (n = 63,998) with the risk in the general population (n = 203,276) reported an elevated risk of AD for gastrectomy patients. Moreover, the risk was even more increased in patients with a total gastrectomy (adjusted hazard ratio: 1.39, 95% confidence interval 01.25–1.54). Interestingly, total gastrectomy patients, which were continually supplemented with vitamin B12, had a reduced AD risk compared to the control (adjusted hazard ratio: 0.71, 95% confidence interval 0.54–0.92) [214].

**Table 3:** Diseases that are linked to vitamin B12 homeostasis and their association with AD.

Link to Vitamin B12		Link to Alzheimer’s Disease	
Author	Main Finding	Author	Main Finding
Inflammatory Bowel Disease (IBD)			
Weisshof et al. (2015) [198]	Micronutrient deficiencies are common (>50%) in patients with IBD with vitamin B12 deficiency belonging to the most common ones.	Zingel et al. (2021) [203]	This study analyzing 3850 patients with an initial diagnosis of inflammatory bowel diseases (IBD; Crohn’s Disease, ulcerative colitis) and 3850 patients without IBD reported that IBD is associated with a 1.22-fold increase in the risk of developing dementia.
Yakut et al. (2010) [199]	Patients with Crohn’s disease common have a serum vitamin B12 deficiency.	Zhan et al. (2021) [204]	An increase in the risk of developing AD was reported in IBD patients in a 16-year longitudinal study including 1742 patients with IBD.
Bermejo et al. (2013) [200]	15.6% (95% CI 9.7–20%) of patients with Crohn’s disease suffer from vitamin B12 deficiency.		
Park et al. (2021) [201]	Crohn’s disease patients are more often deficient in micronutrients like vitamin B12.		
Ward et al. (2015) [202]	The prevalence of vitamin B12 deficiency is common in patients with Crohn’s disease.		
**Gastritis**			
Porter et al. (2021) [207]	Atrophic gastritis was associated with significantly lower serum total vitamin B12 levels and higher prevalence of vitamin B12 deficiency.	Li et al. (2018) [210]	The risk of dementia and AD is increased in patients with many types of autoimmune disorders, like pernicious anemia.
Green (2017) [205]	Pernicious anemia (autoimmune gastritis) is a cause of vitamin B12 deficiency.	Metzler et al. (1991) [212]	Specific clinical entities of a vitamin B12 deficiency include, amongst others, dementia.
Sipponen et al. (2003) [208]	Association of low vitamin B12 serum levels and atrophic gastritis in an elderly male cohort.	Kountouras et al. (2006) [213]	There is a link between an infection with *Helicobacter pylori* and Alzheimer’s disease.

Regarding medications that are associated with a vitamin B12 deficiency, proton pump inhibitors (PPI) are of special interest since they are discussed to be overused. PPIs are commonly prescribed for the treatment of, for example, gastroesophageal reflux disease, reflux esophagitis, gastric and duodenal ulcers, and others. A study in the U.S. ambulatory setting from 2002 until 2009 reported a significant increase in the use of PPIs in general, from 4.0% to 9.2%. Moreover, the highest significant increase was found for the PPI omeprazole (0.9% in 2020 to 3.9% in 2009), which was included in this study next to esomeprazole and pantoprazole [215]. PPIs should be used carefully since they are known to increase the gastric pH into the alkaline milieu, which result in impaired pepsin activation and further protein-bound vitamin B12 malabsorption [216,217,218,219]. In line with this adverse effect, a large population-based study reported a long-term exposure to PPIs of two or more years to be associated with an elevated vitamin B12 deficiency risk (odds ratio, 1.65 and 95% confidence interval, 1.58–1.73). Moreover, the authors reported that the strength of this association depends on the used dosage [220]. These findings are in line with an early study including ten healthy, male volunteers between the age of 22 to 50 years, who were randomly treated with 20 mg or 40 mg omeprazole per day for two weeks. The subsequent evaluation of protein-bound cyanocobalamin (vitamin B12) absorption showed significant dose-dependent decreases in both groups (20 mg omeprazole: from 3.2% to 0.9%, *p* = 0.031; 40 mg omeprazole: 3.4% to 0.4%, *p* < 0.05) [221]. Additionally, a case report from 2002 of a 78-year-old nonvegetarian white woman showed a malabsorption of dietary protein-bound vitamin B12 and vitamin B12 deficiency because of PPI usage for over four years [222].

Based on these adverse effects of PPIs on vitamin B12 uptake and their increased use in the last years, an examination of the potential cognitive impact of PPIs has become a subject of current studies. A randomized controlled trial including sixty healthy volunteers examined the neuropsychological association of the PPIs omeprazole, lansoprazole, pantoprazole, rabeprazole and esomeprazole, with cognitive functions evaluated by five computerized neuropsychological tests of the Cambridge Neuropsychological Test Automated Battery. Visual memory, attention, executive function as well as planning function were measured at the beginning of the study and on Day 7. The results showed that all analyzed PPIs affected cognition in a negative way, with varying degrees of influence between the single PPIs [223]. The outcomes of clinical studies dealing with an association between the use of PPIs and the risk of dementia and AD are heterogenous, as reviewed in a recent meta-analysis involving ten independent studies with more than 600,000 patients [224]. Besides studies resulting in the finding that vitamin B12 deficiency is not associated with an increased risk for dementia or AD [225,226], several clinical investigations reported opposite findings. An observational study from 2016, involving more than 73,000 dementia-free participants older than 75 years, reported a significantly elevated risk of incident dementia in patients using PPIs compared to non-medicated patients (1.44, with 95% confidence interval 1.36–1.52, *p* < 0.001) [227]. These data are in line with previous findings resulting from a longitudinal, multicenter cohort study of more than 3000 community-dwelling persons aged ≥75 years. PPI medication resulted in significantly increased risk of any dementia and AD in comparison to nonusers [228]. Especially long-time usage of PPIs seems to be directly associated with the onset of dementia, as reviewed in [229]. Based on this heterogeneity among studies published so far, the ongoing examination of this potential link is the aim of the current research. Besides the inhomogeneous outcomes of clinical trials, some preclinical studies reported additional mechanisms, next to the caused vitamin B12 deficiency, for the neurological effects of PPIs, which can cross the blood–brain barrier. For example, an interaction with tau protein, influencing the neuronal microenvironment or elevating levels of neurotoxic Aβ, has been described [230]. A further mechanism by which PPIs may increase the risk of dementia, besides causing a deficiency in vitamin B12, was reported recently. An in silico docking study provides evidence that a PPI is a selective inhibitor of choline-acetyltransferase, and this might explain its association with an increased risk of dementia [231]. Based on these findings, the risks and benefits of prescribing PPIs as medication should be balanced individually.

## 5. Veganism/Vegetarianism, Vitamin B12 Levels and AD

Besides the above-mentioned diseases and medications, which can cause a deficiency of vitamin B12 due to a malabsorption, also a low or inadequate dietary intake of this vitamin from animal-sourced foods has been described as a common explanation for a poor vitamin B12 status. In this context, numerous recent observational and clinical studies reported uniformly that vegan and vegetarian diets are strongly associated with a vitamin B12 deficiency [232,233,234,235,236,237,238]. While vegetarian diets exclude animal foods or parts of them, vegan diets exclude animal and all their by-products/derivatives. Reasons for such a kind of diet are multiple and include ethical, spiritual, religious, low socioeconomic status, animal welfare or environmental reasons. In this context, it is suggested to include levels of circulating holotranscobalamin II, which is the bioactive B12 fraction, and total homocysteine, which is a parameter of the metabolic ability, besides serum vitamin B12 concentration to evaluate an individual’s vitamin B12 status [239].

A recent systematic review, which included 12 cohorts and 36 cross-sectional studies, evaluated the adequacy and the micro- and macronutrient intake of vegan diets. The authors reported a lower intake of protein, vitamins (B2, B3, B12 and D), iodine, zinc, calcium, potassium and selenium. Especially the intake of vitamin B12 was significantly reduced from the recommended 2.4 µg to 0.24–0.49 µg in veganism compared to other diet types in this study [240]. Moreover, two randomized controlled trials examining the influences of vegan, vegetarian or Mediterranean diets were recently performed. The first one aimed to investigate the influence of low-calorie lacto-ovo vegetarian in comparison to Mediterranean diets on body weight and the cardiovascular risk in overweight 118 omnivores over three months as the primary outcome. The authors reported significant differences in vitamin B12 levels of 32.32 pg/mL (*p* < 0.01) in end-of-diet values between participants randomly assigned to a vegetarian diet (decrease in vitamin B12 during the study) compared to Mediterranean diet (increased vitamin B12 concentrations at the end of the study) [241]. The second study, which was published in 2019, assigned 53 healthy omnivore participants randomly to a controlled vegan diet without supplements or to a meat-rich diet for four weeks and investigated the vitamin B12 status after this short-term intervention by determining the serum vitamin B12, holotranscobalamin, methylmalonic acid and total plasma homocysteine. Plasma holotranscobalamin was significantly reduced in the vegan diet-treated group compared to the meat-rich group and a lower serum vitamin B12 concentration was found. Additionally, methylmalonic acid and total plasma homocysteine were not changed after this intervention time [242]. Taken together, these studies reported homogenously a causal link between vegan or vegetarian diet patterns and an insufficient supply with the essential micronutrient vitamin B12.

Since the only known naturally source of vitamin B12 are animal food products (meat, poultry, fish, egg, milk, etc.) and the general absence of this vitamin in plant foods because there are no cobalamin-dependent enzymes in plants, strict vegetarians and vegans are advised to supplement vitamin B12 to avoid a deficiency [243,244,245]. Several B12 plant-based food sources were reported on over the last years, for example, Mankai plant [246], seaweed [247], Hippophae rhamnoides, Elymus, Inula helenium [248], some algal species [249] as well as next-generation nutritionally fortified plant-based milk substitutes [250]. One caveat that must be mentioned in this context, is that such natural sources often contain biological inactive vitamin B12 analogues. Methods used to assess the bioavailability of vitamin B12, and the technologies suggested to enhance its absorption, are reviewed in [251]. In a randomized controlled trial, vegans and vegetarians with marginal vitamin B12 deficiency were supplemented with either 350 µg vitamin B12 per week (low dose) or 2000 µg per week (high dose) sublingual for twelve weeks. The outcomes showed no differences in the abilities of both intervention conditions to restore the serum vitamin B12 concentrations and improve the levels of the related metabolic blood markers, and the authors suggested a low dose for nutritional adequacy [252].

As summarized earlier in this review and as homogenously described in the literature, a vitamin B12 deficiency is closely linked to an increased risk of neurodegenerative diseases, such as AD. Considering this link, adequate levels of vitamin B12 are very important in individuals who follow a vegan or vegetarian diet. Moreover, the question arises if there is also a causal link between plant-based diets and cognitive function. Up to now there are no (interventional) studies reporting such a causal link or possible underlying mechanisms [253]. Therefore, one might speculate if the known and common deficit of vitamin B12 in vegans and vegetarians should be weighted more than the positive nutritional aspects that are associated with an animal product-free lifestyle.

On the one hand, most vegans or vegetarians not supplementing micronutrients are affected by a vitamin B12 deficiency, for which the negative aspects regarding AD have been summarized before. On the other hand, plant-based diets are accompanied by a healthy blood lipid profile, for example, due to low levels of saturated fats or cholesterols. Moreover, they are enriched in dietary fiber, flavonoids, folic acid, magnesium or vitamin C, and may be advocated to control energy, as described in a recent comparative study [254].

Taking into consideration that an AD pathology is strongly interconnected with diabetes, obesity, insulin resistance or cardiovascular diseases, preventing strategies including nutritional interventions are discussed as beneficial in AD prevention. Especially plant-based diets with a high intake of for example omega-3 fatty acids or antioxidants and simultaneously reduced intake of saturated fatty acids or proteins derived from animals are favorable, as reviewed in [255]. In contrast, the consumption of red meat was recently shown to be associated with the risk of cognitive impairments in a cohort study including more than 16,000 participants. The intake of meat was measured in the midlife age of the participants (45–74 years) and the risk of cognitive impairment was detected in later life (61–96 years). The authors reported an increased risk of cognitive impairment associated with the highest quartile of red meat intake compared to the lowest quartile. Interestingly, a diet focused on fish showed an association with lowered risks of cognitive impairments [256]. These findings are in line with the results of an earlier (2015) longitudinal study in individuals aged 65 years and older, which reported that a Western dietary pattern (more than seven times a week consumption of meat/poultry; less than four times a week consumption of fish; less than two times a week consumption of beans and legumes; and less than ten times a week consumption of fruits and vegetables) significantly elevated the risk of cognitive decline over eight years (adjusted odds ratio = 4.35, 95% CI = 1.52–12.50, *p* < 0.05) [257].

On a biochemical level, more common risk factors, such as plasma total cholesterol, low-density lipoprotein cholesterol or triacylglycerol levels, have been reported in omnivores eating both plant- and animal-based diets. These risk factors are accompanied by further risky conditions, such as, for example, an increased body mass index or blood pressure [258]. In this context, a vegetarian diet might be beneficial in the prevention of numerous diseases such as hypertension, renal diseases, cardiovascular or dementia, to mention only a few [259]. Furthermore, not only on pathological conditions and diseases but also in the process of healthy aging do plant-based diets seem to be favorable, as reviewed in [260].

In line with these beneficial properties of a plant-based diet but also taking the deficiency of vitamin B12 into consideration, a recent study of adult Canadians recommended a balanced diet of plant- and animal-based protein foods as a healthy nutritional approach [261]. Along with this, it is recommended and important to sensitize especially vegans or vegetarians to be aware of the risk of potential dietary deficiencies, for example, by providing nutritional guidance. This seems to have been met with widespread approval, as a recent cross-sectional study examining the macro- and micronutrient status of vegans reported. The authors reported similarly sufficient vitamin B12 concentrations in vegans and non-vegans and suppose a high rate of supplementation as a possible reason for this finding [262].

## 6. Conclusions

This review focused on the biochemical pathways involved in AD, which are known to be affected by vitamin B12, by summarizing the recent cell culture, animal and clinical studies.

On a molecular level, animal studies demonstrated the influence of vitamin B12 on Aβ generation via β- and γ-secretase cleavage and moreover vitamin B12-dependent alterations of the homocysteine cycle and DNA methylation of BACE1 and PSEN1 promotors. Further, these studies could show that a supplementation of vitamin B12 exerts positive effects with respect to AD pathology, both in transgenic AD models and in wildtype animals. In line with this, cell culture and ex vivo studies provided further evidence for the protective effects of vitamin B12. These are linked to amyloid formation and fibrillization, epigenetic modifications, tau fibrillization, synaptogenesis of neuronal membranes, oxidative stress and cholesterol synthesis. A detailed overview of the proposed beneficial properties of vitamin B12 with respect to amyloid and tau pathology in AD is given in Figure 3.

Clinical studies showed homogenously that vitamin B12 in combination with further representatives of the B vitamin family or alone have beneficial effects on cognitive function, inflammation and brain atrophy in elderly adults without cognitive decline or in mild cognitive impairment patients. Studies dealing with patients suffering from AD found reduced vitamin B12 plasma levels compared to healthy controls. Moreover, supplementation of B vitamins was reported to improve cognitive functions in numerous (randomized) clinical trials.

Interestingly, there are diseases, such as inflammatory bowel disease or gastritis, medications (for example proton pump inhibitors) and surgical interventions (total or partial gastrectomy) that are known to be associated with a vitamin B12 deficiency, and which could be linked to an increased risk of dementia or worse cognitive performance.

Besides these medications or diseases, also a low or inadequate dietary intake of vitamin B12 from animal-based food can be a reason for vitamin B12 hypovitaminosis. Based on this, it is important and recommended to inform vegans and vegetarians to be aware of the risk of their potential dietary deficits. Further research could be to examine the association and molecular mechanisms between a plant-based diet and cognitive function.
